# Synergistic Effect of VS_**2**_/MoS_**2**_ as an Electrocatalyst for Accelerating Polysulfide Conversion in Lithium–Sulfur Batteries

**DOI:** 10.1021/acsami.5c12948

**Published:** 2025-09-25

**Authors:** Thilini Boteju, Abinaya Sivakumaran, Sathish Ponnurangam, Venkataraman Thangadurai

**Affiliations:** † Department of Chemistry, 7486 University of Calgary, Calgary, Alberta T2N 1N4, Canada; ‡ Department of Chemical and Petroleum Engineering, University of Calgary, Calgary, Alberta T2N 1N4, Canada; § School of Chemistry, University of St Andrews, North Haugh, St Andrews KY16 9ST, United Kingdom

**Keywords:** electrocatalyst, lithium−sulfur battery, heterostructure, transition metal sulfides, 2D materials, battery performance, heterocatalyst, density functional theory

## Abstract

The shuttle effect of soluble lithium polysulfides (LiPSs) poses a formidable challenge that severely compromises the electrochemical performance of lithium–sulfur (Li–S) batteries. This study introduces a unique lamellar stacked VS_2_/MoS_2_ nanoflower structure, prepared using a simple one-step hydrothermal synthesis method, to reduce the polysulfide shuttle effect in Li–S batteries. VS_2_/MoS_2_ synergistically boosts LiPS conversion, combining VS_2_’s high conductivity with the catalytic activity of MoS_2_, as confirmed by density functional theory (DFT) calculations. Electrochemical testing demonstrated excellent performance for VS_2_/MoS_2_@S cathodes. It delivers an initial discharge-specific capacity of 1353 mAh g^–1^ at 0.1 C, and at 1 C, the capacity remains as high as 925 mAh g^–1^. At 0.2 C, the initial discharge-specific capacity is 1299 mAh g^–1^, and the capacity retention rate reaches 55% after 500 cycles. This study provides valuable insights into designing and engineering high-performance heterostructures to enhance the adsorption of LiPSs and improve the reaction kinetics in Li–S batteries.

## Graphical abstract

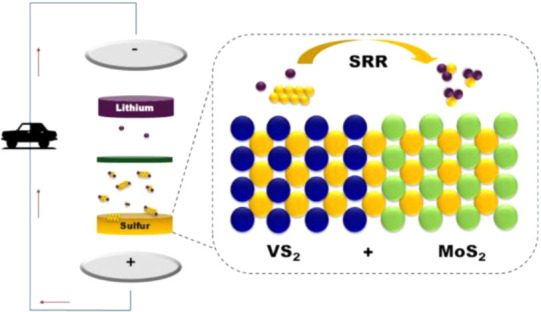

## Introduction

Lithium–sulfur (Li–S) batteries are promising next-generation energy storage devices, offering a high theoretical specific energy density of 2,600 Wh kg^–1^, the natural abundance of sulfur, and an environmentally friendly nature.^1–3^ However, several fundamental challenges impede their practical applications. First, elemental sulfur is inherently insulating, which limits its electrochemical performance.^4^ Second, during the charge–discharge process, long-chain lithium polysulfides (LiPSs) dissolve from the sulfur cathode and diffuse to the lithium anode, a phenomenon known as the “shuttle effect.” This effect results in the loss of active sulfur and rapid capacity decay.^5^ Third, the sulfur reduction reaction (SRR) during the discharge process is slow due to the insulating nature of sulfur and the complex 16-electron redox pathway, further hindering overall battery performance.^6^ These challenges limit the cycling stability and energy density of Li–S batteries, ultimately obstructing their commercialization.^7–9^ To overcome these issues, significant efforts have been directed toward exploring electrocatalysts that can chemically anchor LiPSs and accelerate the conversion reaction between LiPSs and Li_2_S.^10^ These electrocatalysts include metal oxides,^11^ metal sulfides,^12^ metal carbides,^13^ metal nitrides,^14^ single-atom catalysts,^15–17^ MXenes,^18^ MOFs^19^ and heterostructures.^20^

Transition metal (TM)-containing materials can interact with LiPSs through polar–polar interactions. This interaction leads to enhanced adsorption capacity and accelerates redox kinetics, effectively suppressing the detrimental shuttle effect of LiPSs in electrochemical processes. Particularly, transition metal sulfides (TMSs) are considered attractive electrocatalysts for Li–S batteries due to several advantages, including higher stability, strong sulfiphilic properties toward LiPSs, and low lithiation potential vs the lithium anode, which does not overlap with the operating potential range of Li–S batteries.^21^ TMSs exhibit a distinctive layered structure comprising a plane of transition metal atoms (M) intercalated between two hexagonal sulfur planes. This layered configuration, in which covalently bonded planes are linked by weak van der Waals forces, endows TMSs with several advantageous properties. These include the ability to precisely control layer thickness, a high surface-to-volume ratio, and facile surface modification.^22^

Recent advancements in surface engineering, involving the combination of two different TM-containing materials, have gained significant attention for developing heterostructure electrocatalysts in Li–S batteries.^23,24^ These heterostructures may exhibit unique properties that differ significantly from those of their constituent components.^25,26^ Zhou et al. constructed a TiO_2_/TiN heterostructure by combining highly absorptive TiO_2_ and highly conductive TiN, achieving a compromise in efficiency between adsorption and electrocatalysis.^27^ Li et al. demonstrated the synergistic effect of vacancy engineering and heterostructures in material surfaces using a nitrogen-doped graphene/SnS_2_/TiO_2_ nanocomposite to improve the electrochemical performance of Li–S batteries.^28^ They showed a higher initial discharge capacity of 1064 mA h g^–1^ at 0.5 C and a higher capacity retention rate of 68% after 500 cycles at 0.5 C. Zhang et al. demonstrated that the Mo-NH MOF monolayer improves LiPS anchoring through the formation of additional π-bonds, resulting from d-p orbital hybridization between metal d-orbitals and sulfur p-orbitals, and a partially filled antibonding state.^19^

Here, we report a heterostructure electrocatalyst combining two-dimensional (2D) VS_2_ and MoS_2_ structures. MoS_2_ is composed of monolayers stacked with a 6.5 Å gap between two consecutive layers, held together by weak van der Waals forces, and S–Mo–S covalent bonds connecting Mo and S atoms.^29^ MoS_2_ can effectively catalyze the SRR; however, its low conductivity and poor active site density limit the catalytic efficiency of the conversion reaction.^30^ VS_2_, on the other hand, has a hexagonal structure similar to that of MoS_2_ and consists of three S–V–S layers stacked by van der Waals interactions, separated by 5.76 Å. VS_2_ offers intrinsic metallic and highly conductive characteristics with partially filled bands at the Fermi level and a high surface area, but its stability and synthesis can be challenging.^31,32^ Hybrid VS_2_/MoS_2_ is expected to enhance the catalytic activity, improve the electrical conductivity, and provide superior stability. Density functional theory (DFT) calculations were conducted to examine the stability of pristine VS_2_, MoS_2_, and the VS_2_/MoS_2_ heterostructure. Subsequently, electrocatalysts were synthesized by using the hydrothermal approach to assess the anchoring and electrocatalytic activity of the heterostructure relative to pristine VS_2_ and MoS_2_. Sulfur cathodes incorporating pristine and hybrid electrocatalysts were fabricated to evaluate the electrochemical performance of Li–S batteries.

## Experimental Section

### Materials

Ammonium metavanadate (NH_4_VO_3_, ≥ 99%), Sodium Molybdate dihydrate (Na_2_MoO_4_.2H_2_O), ≥ 99.5%), thioacetamide (TAA, 99%), ammonium hydroxide (NH_4_OH, 28–30%), elemental sulfur (99%), activated carbon (<100 mesh), lithium sulfide (99.98%), polyvinylidene fluoride (PVDF, Kynar HSV 900), Super P, *N*-methyl pyrrolidone (NMP, 99%), 1,3-dioxolane (DOL, 99.8%), dimethoxyethane (DME, 99.5%), lithium bis­(trifluoromethane sulfonyl)­imide (LiTFSI, 99.99%), and lithium nitrate (LiNO_3_,99.99%) were purchased from Sigma-Aldrich. All solutions were freshly prepared by using deionized (DI) water.

### Synthesis of VS_2_, MoS_2_, and VS_2_/MoS_2_ Heterostructure

In a typical synthesis, 30 mL of deionized water and 2 mL of ammonia were stirred at room temperature to obtain a homogeneous solution. Then, 0.232 g of NH_4_VO_3_ and 0.242 g of Na_2_MoO_4_.2H_2_O were added to the above solution and sonicated in ice-cold water for 30 min. Finally, 2.254 g of thioacetamide was added to the mixture, and the mixture was stirred overnight. The hydrothermal reaction was performed at 180 °C for 20 h in a 50 mL Teflon-lined autoclave. The final product was collected by centrifuging and washing three times using DI water and ethanol, and then drying in a vacuum oven at 60 °C for 12 h. MoS_2_ and VS_2_ samples were also synthesized under the same experimental conditions without NH_4_VO_3_ and Na_2_MoO_4_.2H_2_O, respectively.

### Material Characterization

Powder X-ray diffraction patterns (PXRD) were obtained using a Bruker D8 Advance diffractometer with Cu K_α_ radiation of λ = 1.5418 Å (40 kV, 40 mA). The data were collected over the 2θ range of 10°–80° with a step size of 0.02° at room temperature. X-ray photoelectron spectra (XPS) were collected using a Thermo-Fisher Escalab QXi XPS with Al K_α_ radiation and analyzed using CasaXPS software.^33^ The morphology of the materials was observed using a Thermo Fisher Scientific Phenom G6 Pro scanning electron microscope (SEM). Transmission electron microscope (TEM) images and energy-dispersive X-ray spectroscopy (EDX) were performed by using the Thermo-Fisher Talos F200X G2 TEM instrument.

### Li_2_S_6_ Absorption Tests

Elemental sulfur and Li_2_S were stirred for 48 h at room temperature in a 5:1 molar ratio in 10 mL of DOL/DME (1:1 v/v) inside an Ar-filled glovebox to make Li_2_S_6_ solution (0.5 M). Li_2_S_6_ solution was then diluted to 3 mM. 40 mg of each electrocatalytic material were placed in 3 mL of DME and DOL solution. All electrocatalysts were heated at 80 °C under vacuum for 12 h before the experiment. To observe the absorption capability, 3 mM Li_2_S_6_ was added to all electrocatalytic samples in the glovebox. After 15 h, 2 mL of supernatant solution from each electrocatalytic sample was collected into a sealed tube for UV–visible spectroscopy analysis. UV–vis analysis was performed by using an Agilent 60 UV–vis instrument. LiPS-adsorbed catalysts were dried inside the glovebox for XPS analysis.

### Li–S Cell Performance Measurements

The electrochemical performances were tested by using CR2032 coin cells. The prepared electrocatalysts were mixed with sulfur and activated carbon (4:4:2 mass ratio) and heated at 150 °C for 6 h under an N_2_ atmosphere. Activated carbon enhances the electronic conductivity of the cathode to compensate for the insulating nature of elemental sulfur, provides mechanical flexibility and structural support, facilitates charge transfer, and maintains electrode integrity over prolonged cycling. The resulting composite, Super P, and PVDF binder were dissolved in NMP at an 8:1:1 ratio to form a black slurry. The slurry was then coated onto aluminum foil current collectors and dried in a vacuum oven at 60 °C for 20 h. The average loading for sulfur cathodes was about 1.7 mg/cm^2^. The electrolyte consisted of 1.0 M LiTFSI in a 1:1 mixture of DOL and DME, with 0.1 M LiNO_3_ as an additive. Elemental lithium metal was used as the anode. The coin cells were assembled under an Ar atmosphere inside a glovebox. The charging–discharging performances of the coin cells were analyzed using a Neware battery testing station with a potential window of 1.5 V to 3.0 V. Cyclic voltammetry (CV) and electrochemical impedance spectroscopy (EIS) were performed using the EC-Lab software in a BioLogic potentiostat.

### Density Functional Theory Calculations

The first-principles calculations were performed by the Vienna Ab initio Simulation Package (VASP) with the projector-augmented wave.^34–36^ The cutoff energy was set to 500 eV, and a Monkhorst–Pack k-point grid of 3 × 3 × 1 was used for the calculations.^37–39^ The vdW correction proposed by Grimme (DFT-D2) was applied to account for long-range interactions, and a 15 Å vacuum layer was included to prevent interactions between neighboring images.^40^ All structures were relaxed until the energy (10^–5^ eV) and force (0.02 eV/Å) converged. The DFT + U method, with U values of 3 eV and 3.25 eV for Mo and V, respectively, was employed for the density of states calculations.^41,42^ The results were analyzed using the VASPkit package.^43^

## Results and Discussion

The VS_2_/MoS_2_ heterostructure was constructed using a 3 × 3 × 1 supercell of the 2H phase of monolayer MoS_2_ and VS_2_. The lattice constants calculated for VS_2_ and MoS_2_ monolayers are *a* = *b* = 3.17 Å and *a* = *b* = 3.18 Å, respectively, which align with existing literature.^44,45^ Due to the similar lattice constants of the two monolayers in the hexagonal geometry (2H phase), the VS_2_ monolayer can completely cover the MoS_2_ monolayer with minimal lattice mismatch (∼0.31%). The heterostructures were created by stacking the VS_2_ monolayer on top of the MoS_2_ monolayer. The top and side views of the AA-stacking and AB-stacking configurations are shown in Figure 1a,b. In AA-stacking, the S atom in VS_2_ aligns on top of the S atom in MoS_2_, whereas in AB-stacking, the S atom on VS_2_ is placed on top of the Mo atom of MoS_2_. The binding energies of these two configurations were calculated using

**Figure 1:**
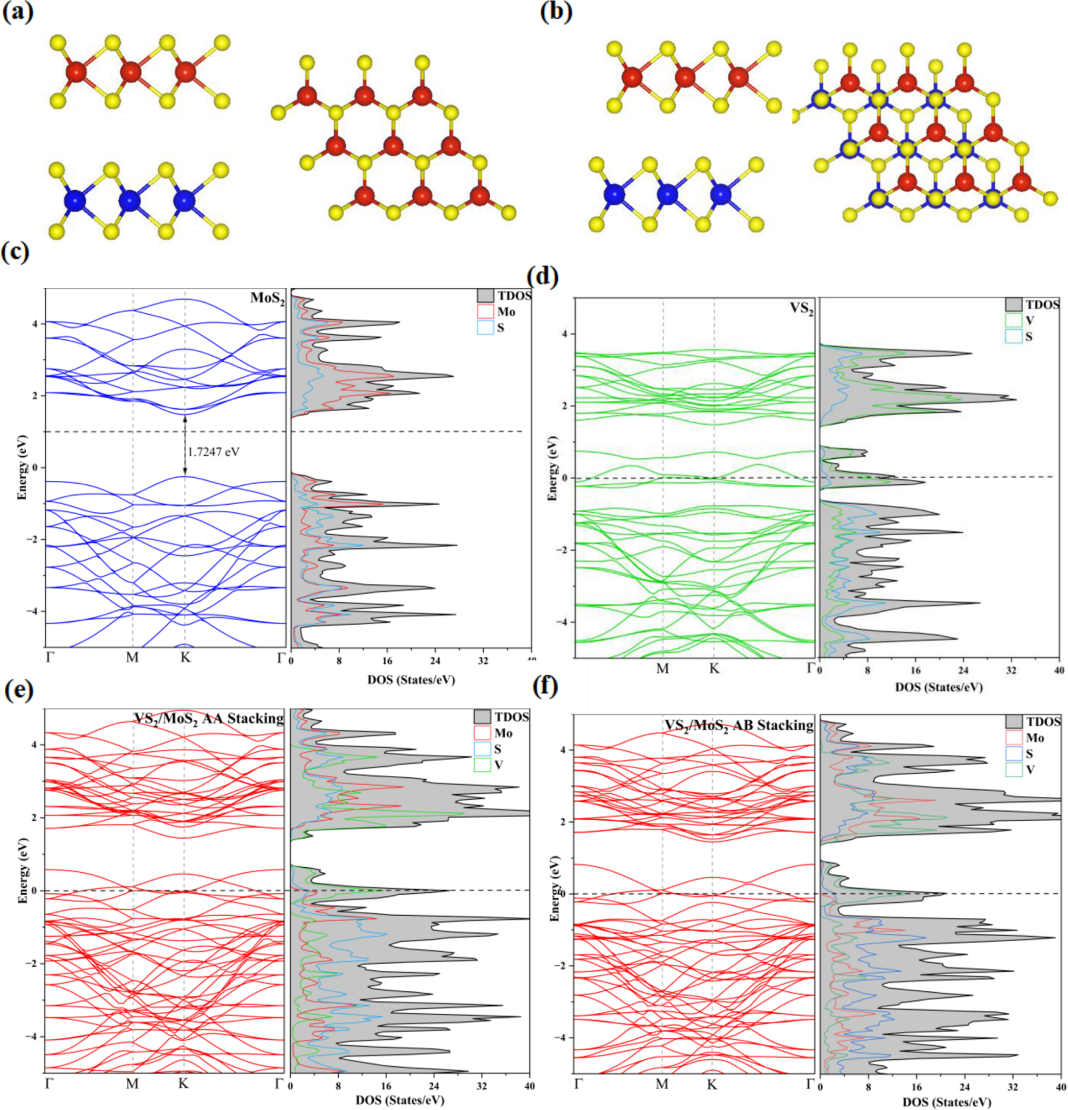
Top and side views of (a) AA stacking and (b) AB stacking. Red and blue spheres represent V and Mo, respectively, while the yellow sphere represents S. (c-f) Electronic band structure and density of states for MoS_2_, VS_2_, and VS_2_/MoS_2_ heterostructure.


1 $$E_{b}=E_{T}-E_{\mathrm{VS}2}-E_{MoS2}$$

where *E*_T_, *E*_VS2_, and *E*_MoS2_ are the optimized energies of the VS_2_/MoS_2_ heterostructure, VS_2_ monolayer, and MoS_2_ monolayer, respectively. The negative binding energies of −1.22 eV for AA-stacking and −1.84 eV for AB-stacking indicate that heterostructure configurations are energetically favorable.^46^ However, apart from the high-symmetry AA- and AB-stacking models, other stacking models (such as random stacking or twisted stacking) and lattice defects may exist in actual materials. These variations can also modify the electronic properties and catalytic activity of the heterostructure electrocatalyst.

Figure 1c–f presents the calculated total band structure and electronic density of states (DOS) of VS_2_, MoS_2_, and VS_2_/MoS_2_ heterostructure, respectively. The band gap of pure MoS_2_ is 1.7090 eV, while pure VS_2_ has no band gap, consistent with previous reports.^47,48^ Nonetheless, the absence of a band gap near the Fermi level in the VS_2_/MoS_2_ heterostructure indicates that the combination of a semiconducting MoS_2_ monolayer and a metallic VS_2_ monolayer produces a metallic composite. This demonstrates that the introduction of VS_2_ leads to a rearrangement of the energy bands, resulting in a significant improvement in the electrical conductivity within the VS_2_/MoS_2_ heterostructure. The density of states calculations further confirm the above results. VS_2_/MoS_2_ heterostructures were synthesized by using a facile one-step hydrothermal method with ammonium metavanadate (NH_4_VO_3_), sodium molybdate tetrahydrate (Na_2_MoO_4_.2H_2_O), and thioacetamide (C_2_H_5_NS) as vanadium, molybdenum, and sulfur sources, respectively. The synthesis process of the VS_2_/MoS_2_ heterostructure is schematically illustrated in Figure 2a.

**Figure 2:**
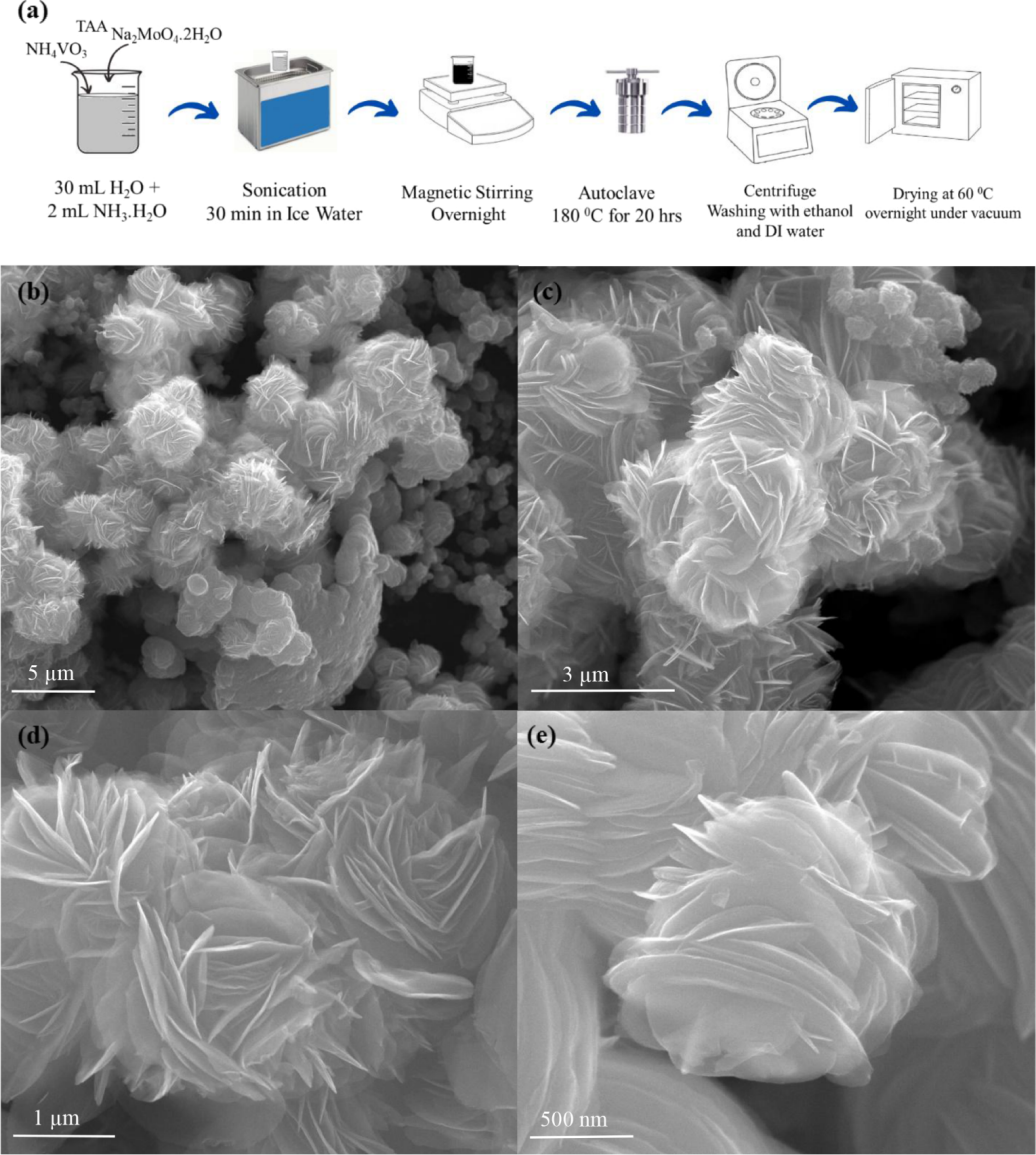
(a) Synthesis procedure; (b–e) SEM images of the VS_2_/MoS_2_ heterostructure observed at different magnifications.

The morphology and elemental composition of the VS_2_/MoS_2_ heterostructure were characterized by using scanning electron microscopy (SEM), energy-dispersive X-ray (EDX) spectroscopy, and transmission electron microscopy (TEM). As shown in Figure S1a–d, VS_2_ consists of flower-like, vertically stacked nanoplates arranged in various orientations, while MoS_2_ consists of nanopetal structures. Figure 2 shows SEM images of the VS_2_/MoS_2_ nanosheets. Low-magnification images (Figure 2b,c) reveal different orientations of the nanosheets in flower-like clusters.

High-resolution images in Figure 2d,e confirm the presence of numerous lamellar-stacked nanosheets oriented in different directions within the VS_2_/MoS_2_ heterostructure. This flower-like structure plays a critical role in accommodating sulfur within the cathode composite. The lamellar-stacked nanosheets offer a high surface area, facilitating sulfur dispersion while mitigating volume expansion during cycling. The layered architecture also enhances electrochemical accessibility and promotes efficient redox kinetics for LiPS conversion.

Figure 3a presents a low-magnification TEM image, providing an overview of the hybrid structure. The selected area electron diffraction (SAED) pattern, shown in Figure 3b, exhibits a hexagonal crystal lattice projected along the (001) axis, confirming the *c*-axis stacking of VS_2_ within the heterostructure. The higher-magnification TEM in Figure 3c–e show a lattice spacing (d) of 0.61 nm, which coincides with the (002) plane of MoS_2_, and the 0.57 nm *d*-spacing corresponds to the (001) plane in VS_2_. The energy-dispersive X-ray (EDX) elemental mapping shows a uniform distribution of V, Mo, and S elements throughout the VS_2_/MoS_2_ heterostructure. For comparison, the elemental distributions of VS_2_ and MoS_2_ are shown in Figure S1e,f.

**Figure 3:**
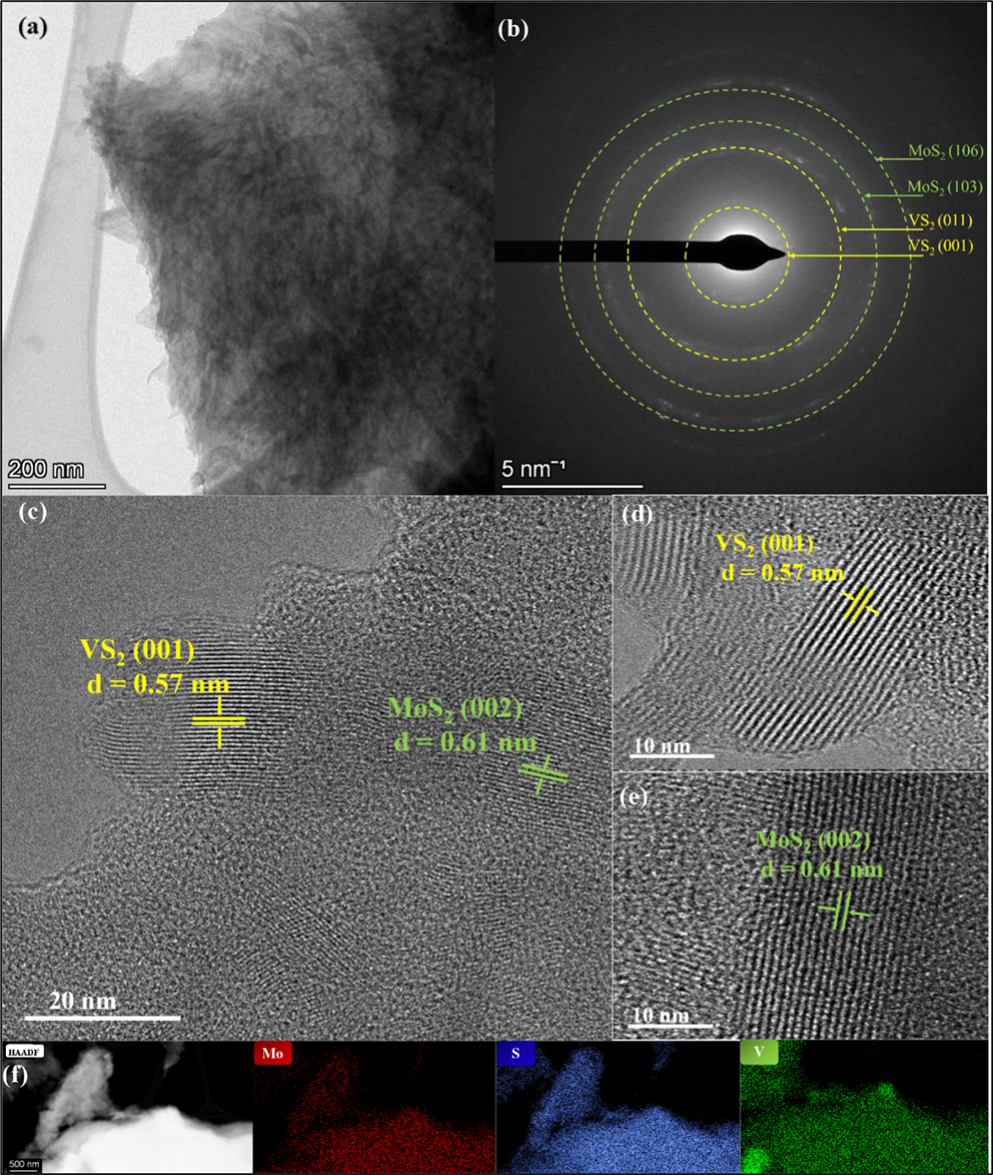
(a) Low-magnification TEM image; (b) SAED patterns; and (c-e) high-resolution TEM (HRTEM) images of VS_2_/MoS_2_ heterostructure (f) High-angle annular dark-field (HAADF) TEM images of VS_2_/MoS_2_ and the elemental mapping of V, Mo, and S.

To further analyze the structure, powder X-ray diffraction (PXRD), Raman spectroscopy, and X-ray photoelectron spectroscopy (XPS) characterization studies were performed. Figure 4a presents the XRD patterns of MoS_2_, VS_2_, and VS_2_/MoS_2_. The XRD pattern of the heterostructure VS_2_/MoS_2_ exhibits characteristic peaks at 15.38°, 35.67°, 45.23°, and 57.82° corresponding to the (001), (011), (012), and (110) crystal planes of the 2H phase of hexagonal VS_2_ according to the standard PDF (#89-1640). The prominent peaks at 14.38°, 33.43°, and 59.06° correspond to the (002), (100), and (110) crystal planes of hexagonal MoS_2_ according to the standard PDF card (#73-1508). VS_2_ and MoS_2_ have the same space group symmetry, denoted by *p*-3m 1. Figure 4b displays the Raman spectra of the VS_2_/MoS_2_ heterostructure, revealing four prominent peaks. Two peaks at 280 and 404 cm^– 1^ correspond to the E_g_ and A_g_ modes of VS_2_, respectively. The other two peaks, at 381 and 407 cm^– 1^, are attributed to the $E_{2}^{1}g$ and A_1g_ modes of MoS_2_.

**Figure 4:**
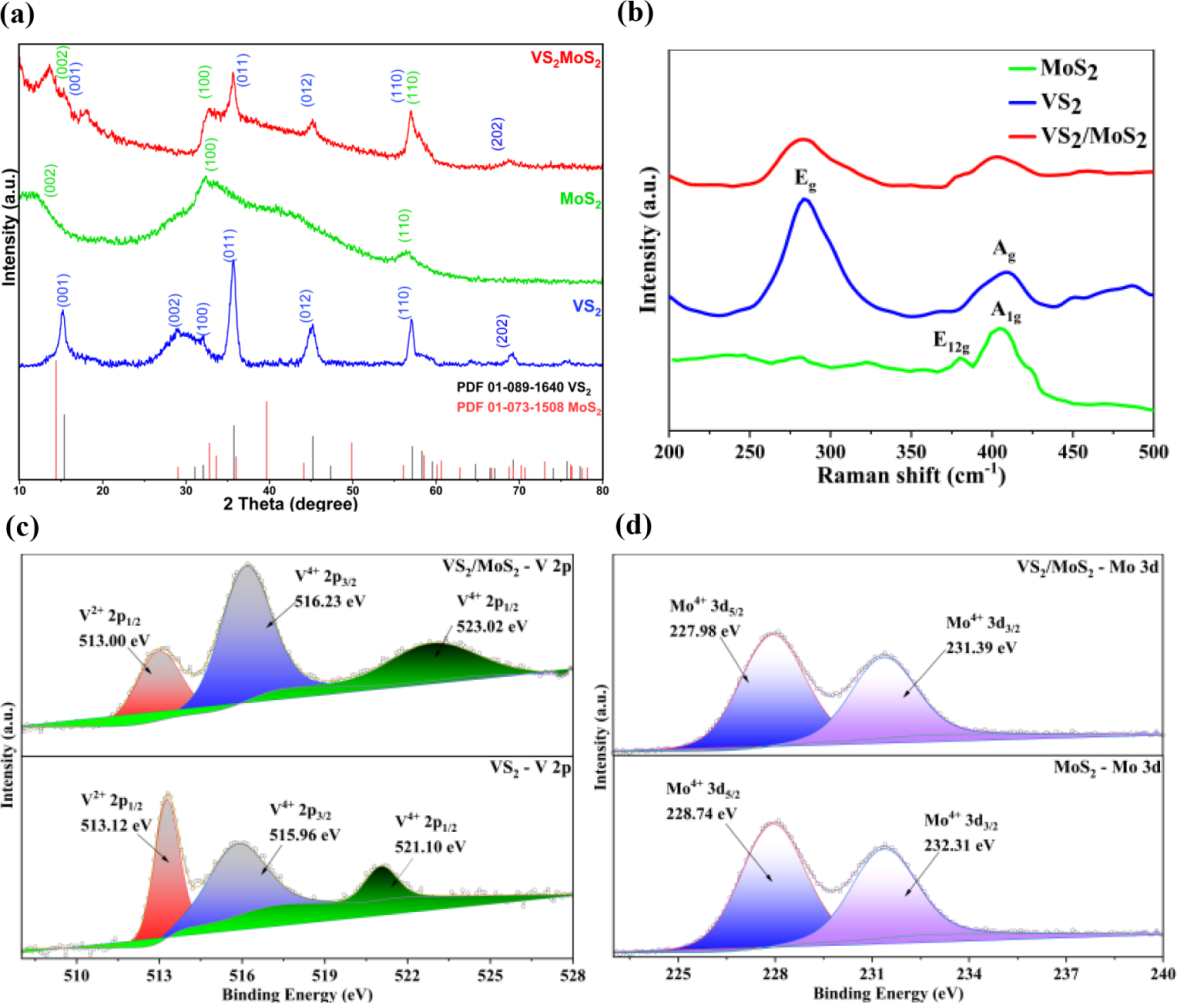
(a) PXRD patterns for VS_2_, MoS_2_, and VS_2_/MoS_2_; (b) Raman spectra of VS_2_, MoS_2_, and VS_2_/MoS_2_; XPS spectra for (c) V 2p and (d) Mo 3d for VS_2_, MoS_2_, and VS_2_/MoS_2_.

X-ray photoelectron spectroscopy (XPS) was performed to analyze the elemental composition and chemical state of the VS_2_/MoS_2_ heterostructure. Figure 4c shows that three characteristic peaks located at 513.0, 516.23, and 523.02 eV are assigned to V^2+^ 2p_1/2_, V^4+^ 2p_3/2_, and V^2+^ 2p_1/2_ orbitals, respectively, indicating a slight shift to lower binding energy compared to V 2p in VS_2_. As shown in Figure 4d, the binding energies of the Mo^4+^ 3d_3/2_ (231.39 eV) and Mo^4+^ 3d_5/2_ (227.98 eV) orbitals confirm the presence of Mo^4+^ in VS_2_/MoS_2_. Interestingly, the Mo^4+^ 3d_3/2_ and Mo^4+^ 3d_5/2_ peaks of pure MoS_2_ have shifted to higher binding energies of 228.74 and 232.31 eV, respectively, compared to VS_2_/MoS_2_. The observed peak shifts demonstrate a strong electronic interaction between MoS_2_ and VS_2_ in the heterostructure, and the electrons may transfer from VS_2_ to MoS_2_.^49,50^

To evaluate the performance of the electrocatalysts, a LiPS adsorption test was conducted using Li_2_S_6_ as a representative example. VS_2_, MoS_2_, and VS_2_/MoS_2_ were added separately into a 3 mM Li_2_S_6_ solution in DME/DOL (v/v = 1:1) mixed solvent in sealed vials. After 15 h, the solution containing VS_2_/MoS_2_ was transparent, while the solutions with VS_2_ and MoS_2_ showed a color decay, as shown in Figure 5a. Figure 5b shows the corresponding UV–vis absorption spectra. An absorbance peak was observed in the 250–350 nm region for all samples due to the presence of S_6_^2–^ in Li_2_S_6_. However, the peak intensity of the solution containing VS_2_/MoS_2_ was much lower than those of the other two solutions, which is consistent with the observed color decay. Figure 5c shows the DFT-calculated binding energies for Li_2_S_6_ on AA- and AB-stacked VS_2_/MoS_2_ configurations. The binding energies show moderate binding values, which confirm the ability of the heterostructure to act as a host material for LiPSs. In addition, XPS analysis was carried out to further understand the interactions between electrocatalysts and polysulfides before and after adsorption. As shown in Figure 5d,e, after Li_2_S_6_ adsorption, the characteristic Mo^4+^ 3d_5/2_ and 3d_3/2_ peaks in VS_2_/MoS_2_ shift toward higher binding energies to 232.43 and 228.18 eV, respectively, indicating electron transfer from Mo atoms to the Li_2_S_6_ molecules. Similarly, V^2+^ 2p_1/2_, V^4+^ 2p_3/2_, and V^4+^ 2p_1/2_ peaks have shifted to higher binding energies of 513.12, 516.20, and 523.04 eV, respectively, confirming the strong chemical adsorption.

**Figure 5:**
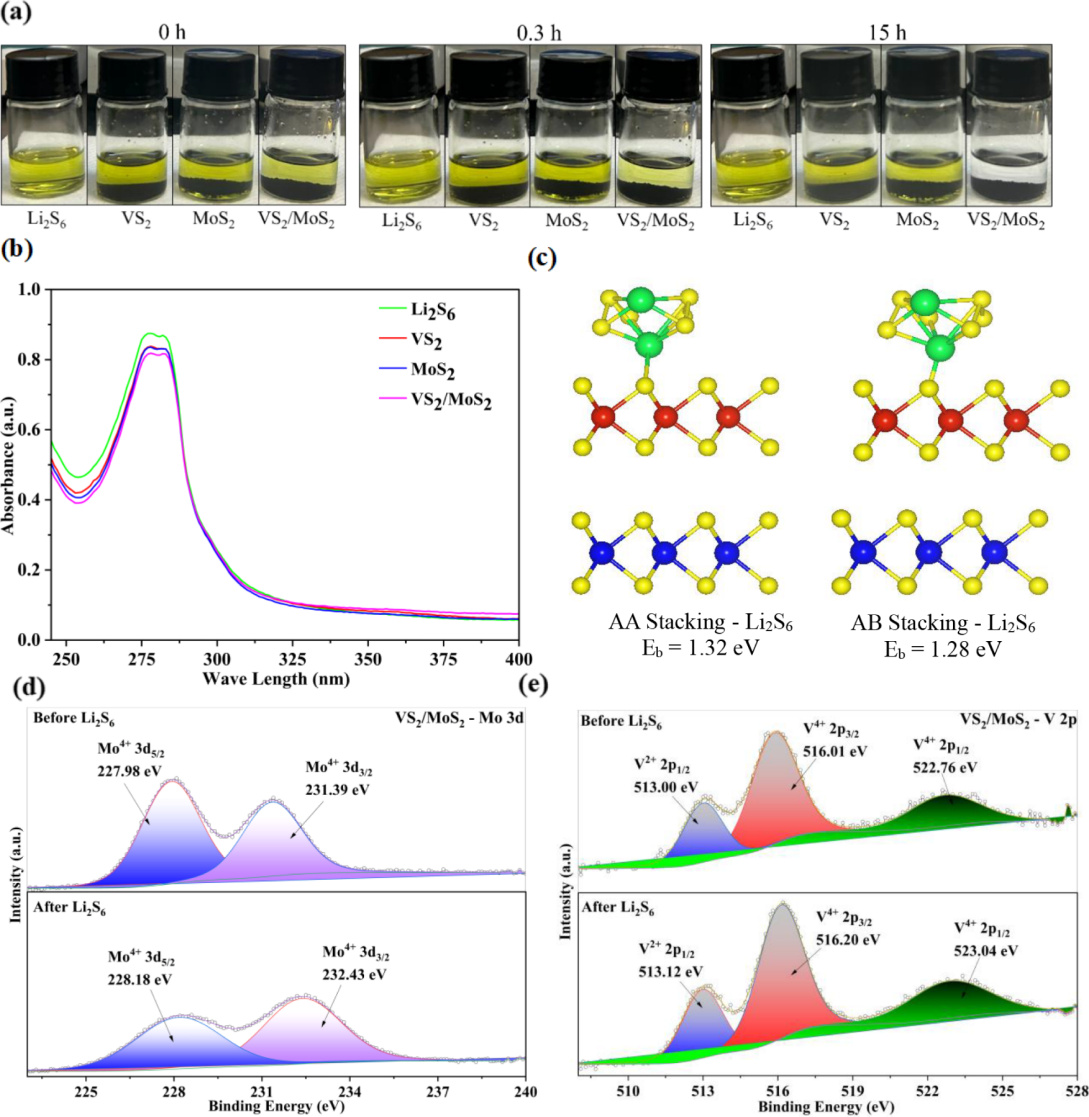
(a) Image of the Li_2_S_6_ adsorption test; (b) UV–vis spectra for blank Li_2_S_6_, VS_2_, MoS_2_, and VS_2_/MoS_2_; (c) DFT-calculated binding energies and the adsorption configurations of Li_2_S_6_ on AA- and AB-stacked VS_2_/MoS_2_; XPS for (d) Mo 3d peak and (e) V 2p peak for VS_2_/MoS_2_ before and after adsorption.

The electrochemical properties of the Li–S cells were investigated by using electrochemical impedance spectroscopy (EIS), cyclic voltammetry (CV), and galvanostatic charge/discharge (GCD) measurements.

The CV measurements at 0.1 mVs^–1^ scan rate were conducted on VS_2_/MoS_2_@S, VS_2_@S, and MoS_2_@S cathodes to assess their electrochemical performance. The CV curves, consistent with prior studies, exhibited characteristic redox peaks.^51^ During the cathodic sweep, two reduction peaks were observed. The first peak, around 2.27 V, corresponds to the reduction of sulfur to long-chain LiPSs (Li_2_Sn, 4 ≤ *n* < 8). The second peak, near 1.92 V, is attributed to the further reduction of these long-chain polysulfides to shorter-chain LiPSs, Li_2_S_2_, and Li_2_S. The anodic sweep showed an oxidation peak at 2.50 V, indicative of the reoxidation of short-chain LiPSs to long-chain LiPSs and, ultimately, elemental sulfur. As depicted in Figure 6a, compared to VS_2_@S and MoS_2_@S cathodes, the VS_2_/MoS_2_@S cathode shows a shift to a higher potential in the reduction peaks and a shift to a lower potential in the oxidation peak, along with the highest peak current response. This can be attributed to the accelerated redox kinetics of LiPSs on VS_2_/MoS_2_@S, which effectively minimizes electrochemical polarization. As shown in Figure 6c, the Tafel slope of VS_2_/MoS_2_@S is 133.24 mV dec^–1^ for the oxidation peak, which is smaller than the Tafel slopes of VS_2_@S (176.92 mV dec^–1^) and MoS_2_@S (206.18 mV dec^–1^). A similar trend was observed in the reduction phase, where the Tafel slope of VS_2_/MoS_2_@S (Figure 6b) is 86.53 mV dec^–1^, which is much smaller than that of VS_2_@S (93.69 mV dec^–1^) and MoS_2_@S (104.88 mV dec^–1^). This electrochemical analysis proves that VS_2_/MoS_2_@S has reduced the redox energy barrier of the SRR, and the cathode active material utilization has improved. Notably, the CV curves of the VS_2_/MoS_2_@S cathode displayed excellent overlap during the second and third cycles and a slight deviation of the oxidation peak in the first cycle, signifying high electrochemical stability, reversibility, and minimal loss of active sulfur (Figure 6d).

**Figure 6:**
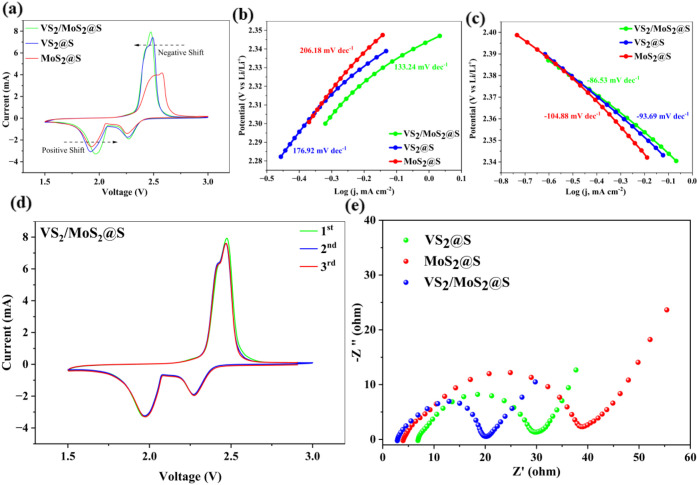
(a) CV curves of VS_2_/MoS_2_@S, VS_2_@S, and MoS_2_@S-containing electrodes swept in the voltage range of 1.5–3.0 V at a scan rate of 0.1 mVs^–1^. Tafel plots for (b) reduction and (c) oxidation processes; (d) CV curve of the first three cycles for VS_2_/MoS_2_@S. (e) EIS plots for VS_2_/MoS_2_@S, VS_2_@S, and MoS_2_@S before cycling.

Electrochemical Impedance Spectroscopy (EIS) before cycling was conducted to compare the cell’s performance after introducing electrocatalysts (Figure 6e). EIS data, when plotted as Nyquist diagrams, commonly show a high-frequency arc followed by a low-frequency linear segment. This pattern implies the existence of both charge transfer resistance (R_ct_) and Li^+^ diffusion resistance (R_Ω_). The R_ct_ values of Li–S batteries with VS_2_, MoS_2_, and VS_2_/MoS_2_ are 20 Ω, 30 Ω, and 15 Ω, respectively. Figure S2 and Table S1 provide the corresponding equivalent circuits and detailed fitting values in the Supporting Information. EIS analysis reveals that incorporating a VS_2_/MoS_2_ heterostructure significantly enhances charge transport within the Li–S battery. This facilitates effective electron transfer at the cathode during the discharge and charge reactions.

Figure 7a exhibits the initial discharge–charge profiles of the VS_2_/MoS_2_@S, VS_2_@S, and MoS_2_@S cathodes at 0.1 C. All cells showed one charge and two discharge plateaus, similar to those in the CV plots in Figure 6a. The charge plateaus correspond to the conversion from Li_2_S_2_/Li_2_S to S_8_. In the discharge profiles, the higher plateau is attributed to the conversion of sulfur to soluble higher-order LiPSs, while the lower plateau corresponds to the reduction of these soluble LiPSs to Li_2_S_2_/Li_2_S.^52^ The VS_2_/MoS_2_@S cathode delivers a significantly higher initial specific discharge capacity of 1353 mAh g^–1^ at 0.1 C compared to the VS_2_@S and MoS_2_@S cathodes, which exhibit much lower initial specific capacities of 1159 mAh g^–1^ and 906 mAh g^–1^, respectively. Figure 7b presents the initial discharge–charge capacities of VS_2_/MoS_2_@S at various current rates. The discharge capacities were measured at 0.1 C, 0.2 C, 0.5 C, 1 C, and 2 C, resulting in values of 1353, 1308, 1272, 925, and 563 mAh g^–1^, respectively. It is evident that with the increase in current density, the polarization intensity increases, leading to a gradual decrease in the discharge capacity. However, even at 2 C, VS_2_/MoS_2_@S showed a higher initial discharge capacity of 563 mAh g^–1^. Figure 7c illustrates the discharge–charge profiles of the VS_2_/MoS_2_@S cathode at 0.1 C for 200 cycles. After 200 cycles, VS_2_/MoS_2_@S retained a discharge capacity of 746 mAh g^–1^ with 55% capacity retention. Figure 7d shows the consecutive cyclic performance for all three cathodes at different current rates, measured for ten cycles at each current in ascending steps from 0.1 C to 2 C and then returning to 0.1 C. Notably, after the rate capability test, the VS_2_/MoS_2_@S showed a capacity of 944 mAh g^–1^ at 0.1 C. The long-term cycling performance of VS_2_/MoS_2_@S cathode at 0.2 C is shown in Figure 7e. After 500 cycles, it retains a capacity of 721 mAh g^–1^ capacity, corresponding to a capacity retention of 55%, indicating good cycling stability. In comparison, the VS_2_@S cathode retains 417 mAh g^–1^ and MoS_2_@S 347 mAh g^–1^ discharge capacity and 39.7% and 36.4% capacity retentions, respectively. This capacity decay may be attributed to the gradual dissolution of LiPSs and incomplete Li_2_S decomposition reactions, which can result in the accumulation of insulating discharge products and a reduced active surface area of the cathode.

**Figure 7:**
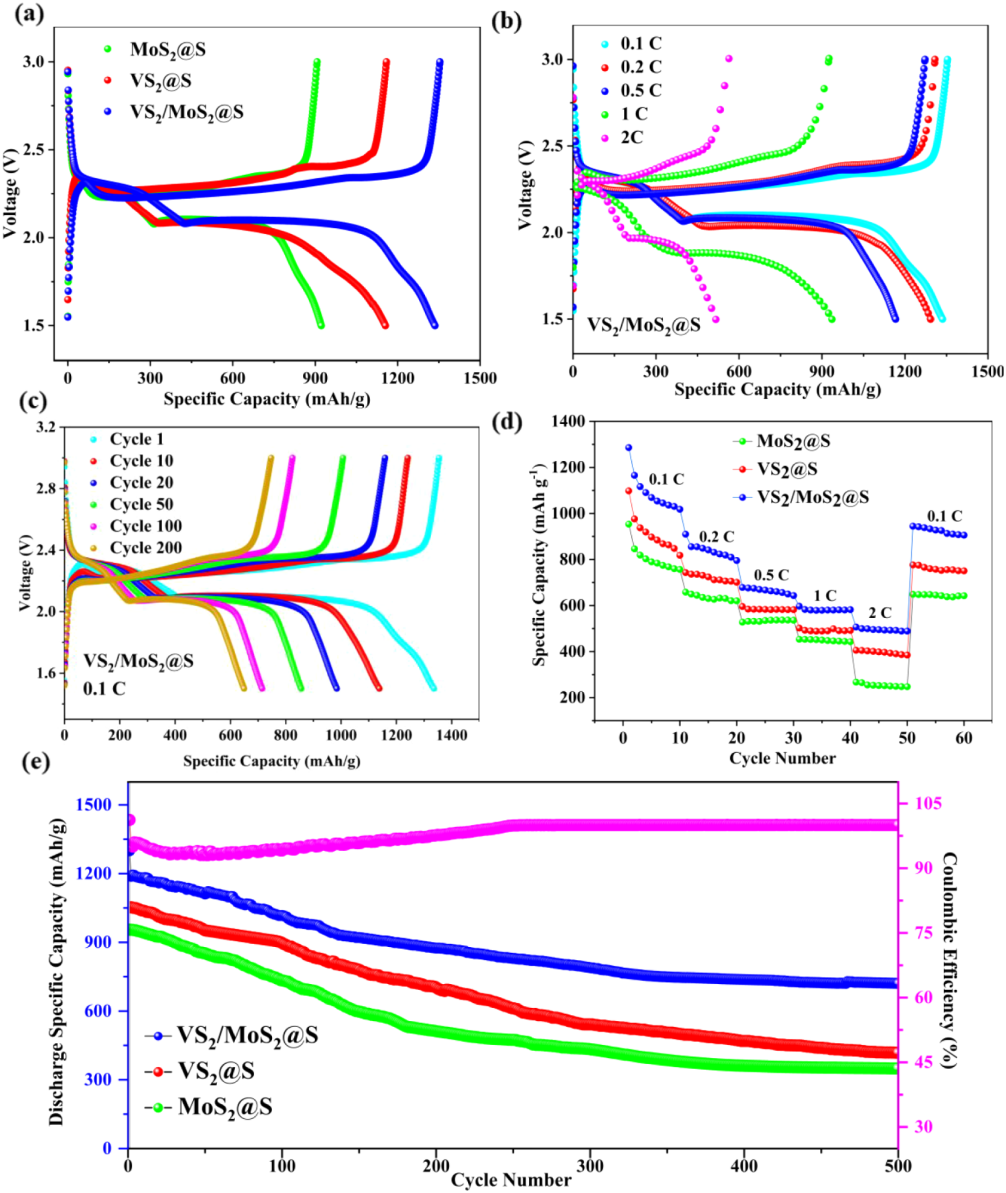
(a) Comparison of initial discharge–charge profiles for VS_2_/MoS_2_@S, VS_2_@S, and MoS_2_@S cathodes at 0.1 C; (b) discharge–charge profiles for VS_2_/MoS_2_@S at various C rates from 0.1 C to 2 C; (c) discharge–charge profiles for VS_2_/MoS_2_@S cathode from cycle 1 to 200 at 0.1 C; (d) rate performance; (e) long-term cycling performance at 0.2 C for 500 cycles.

## Conclusions

This study reports the facile one-step hydrothermal synthesis of a VS_2_/MoS_2_ heterostructure electrocatalyst as a promising sulfur host for Li–S batteries. Detailed first-principles calculations of the heterostructure’s geometry and electronic band structure were performed, revealing that the AB-stacked configuration exhibits greater stability than the AA-stacked configuration based on binding energy calculations. The DFT calculations and LiPS absorption tests reveal that the VS_2_/MoS_2_ heterostructure modifies the electronic band structure, resulting in faster reaction kinetics and increased LiPS adsorption. The synergistic combination of high conductivity in VS_2_ and the strong adsorption and catalytic capabilities of MoS_2_ greatly suppresses the LiPS shuttling, accelerates the redox kinetics, and boosts the electrochemical performance of Li–S batteries. The vertically stacked 2D lamellar structure of VS_2_/MoS_2_ provides abundant active sites and short diffusion paths, improving the ion transport kinetics. Consequently, Li–S batteries employing a VS_2_/MoS_2_@S cathode exhibit significantly improved electrochemical performance compared to those of pristine VS_2_@S and MoS_2_@S cathodes. The VS_2_/MoS_2_@S cathode achieves an initial discharge-specific capacity of 1353 mAh g^–1^ at 0.1 C, maintaining a high capacity of 925 mAh g^–1^ even at 1 C. At 0.2 C, the initial discharge-specific capacity is 1299 mAh g^–1^, with a capacity retention of 55% after 500 cycles. This study highlights the potential of VS_2_/MoS_2_ as an electrocatalyst for high-performance Li–S batteries and offers valuable insights into designing advanced heterogeneous composite catalytic materials.

## Supplementary Material



## Notes

SEM images of pristine MoS_2_ and VS_2_, equivalent circuit diagram used in EIS, charge–discharge profiles, EIS fitting parameters, and literature comparison of electrocatalysts (PDF)

## References

[1] Manthiram A., Fu Y., Su Y. S. (2013). Challenges and Prospects of Lithium-Sulfur Batteries. Acc. Chem. Res..

[2] Wang M., Bai Z., Yang T., Nie C., Xu X., Wang Y., Yang J., Dou S., Wang N. (2022). Advances in High Sulfur
Loading Cathodes for Practical Lithium-Sulfur Batteries. Adv. Energy Mater..

[3] Deng R., Wang M., Yu H., Luo S., Li J., Chu F., Liu B., Wu F. (2022). Recent Advances and Applications
Toward Emerging Lithium–Sulfur Batteries: Working Principles
and Opportunities. Energy Environ. Mater..

[4] Bonnick P., Muldoon J. (2020). The Dr Jekyll and Mr
Hyde of lithium sulfur batteries. Energy &
Environmental Science.

[5] Ren W., Ma W., Zhang S., Tang B. (2019). Recent Advances in Shuttle Effect
Inhibition for Lithium Sulfur Batteries. Energy
Storage Mater..

[6] Zhou L., Danilov D. L., Qiao F., Wang J., Li H., Eichel R. A., Notten P. H. L. (2022). Sulfur Reduction Reaction in Lithium–Sulfur
Batteries: Mechanisms, Catalysts, and Characterization. Adv. Energy Mater..

[7] Huang Y., Lin L., Zhang C., Liu L., Li Y., Qiao Z., Lin J., Wei Q., Wang L., Xie Q. (2022). Recent
Advances and Strategies toward Polysulfides Shuttle Inhibition for
High-Performance Li–S Batteries. Adv.
Sci..

[8] Wang J., Wang H., Jia S., Zhao Q., Zheng Q., Ma Y., Ma T., Li X. (2023). Recent Advances in Inhibiting Shuttle
Effect of Polysulfide in Lithium-Sulfur Batteries. J. Energy Storage.

[9] Zhang Z., Chen C., Xu J., Lin Z., Lin Z. (2022). Nanoporous
Cobalt-Nitrogen-Carbon Catalyst-Based Multifunctional Interlayer for
Enhanced Li-S Battery Performance. ACS Appl.
Energy Mater..

[10] Wang S., Wang Z., Chen F., Peng B., Xu J., Li J., Lv Y., Kang Q., Xia A., Ma L. (2023). Electrocatalysts
in Lithium-Sulfur Batteries. Nano Res..

[11] Seh Z. W., Li W., Cha J. J., Zheng G., Yang Y., McDowell M. T., Hsu P. C., Cui Y. (2013). Sulphur-TiO2 Yolk-Shell Nanoarchitecture
with Internal Void Space for Long-Cycle Lithium-Sulphur Batteries. Nat. Commun..

[12] Boteju T., Abraham A. M., Ponnurangam S., Thangadurai V. (2023). Theoretical
Study on the Role of Solvents in Lithium Polysulfide Anchoring on
Vanadium Disulfide Facets for Lithium-Sulfur Batteries. J. Phys. Chem. C.

[13] Zhou F., Li Z., Luo X., Wu T., Jiang B., Lu L. L., Yao H. B., Antonietti M., Yu S. H. (2018). Low Cost Metal Carbide
Nanocrystals as Binding and Electrocatalytic Sites for High Performance
Li-S Batteries. Nano Lett..

[14] Sun Z., Zhang J., Yin L., Hu G., Fang R., Cheng H. M., Li F. (2017). Conductive Porous Vanadium
Nitride/Graphene
Composite as Chemical Anchor of Polysulfides for Lithium-Sulfur Batteries. Nat. Commun..

[15] Song Y., Zou L., Wei C., Zhou Y., Hu Y. (2023). Single-atom electrocatalysts
for lithium–sulfur chemistry: Design principle, mechanism,
and outlook. Carbon Energy.

[16] Zhang W., Wang S., He C. (2025). New Insights into Axially Asymmetric
Mechanism for Enhanced Anchoring and Catalytic Performance in Lithium–Sulfur
Batteries. J. Colloid Interface Sci..

[17] Sun H., Li X., Chen T., Xia S., Yuan T., Yang J., Pang Y., Zheng S. (2023). In Situ Trapping
Strategy Enables
a High-Loading Ni Single-Atom Catalyst as a Separator Modifier for
a High-Performance Li-S Battery. ACS Appl. Mater.
Interfaces.

[18] Boteju T., Ponnurangam S., Thangadurai V. (2024). MXenes as Effective Sulfur Hosts
and Electrocatalysts to Suppress Lithium Polysulfide Shuttling: A
Computational Study. J. Phys. Chem. C.

[19] Zhang W., He X., He C. (2025). The “d-p Orbital Hybridization”-Guided
Design of Novel Two-Dimensional MOFs with High Anchoring and Catalytic
Capacities in Lithium – Sulfur Batteries. J. Colloid Interface Sci..

[20] Wen Y., Shen Z., Hui J., Zhang H., Zhu Q. (2023). Co/CoSe Junctions
Enable Efficient and Durable Electrocatalytic Conversion of Polysulfides
for High-Performance Li–S Batteries. Adv. Energy Mater..

[21] He J., Manthiram A. (2019). A Review on
the Status and Challenges of Electrocatalysts
in Lithium-Sulfur Batteries. Energy Storage
Mater..

[22] Zhang S., Wang J., Torad N. L., Xia W., Aslam M. A., Kaneti Y. V., Hou Z., Ding Z., Da B., Fatehmulla A. (2020). Rational Design of Nanoporous MoS2/VS2 Heteroarchitecture
for Ultrahigh Performance Ammonia Sensors. Small.

[23] Ye C., Jiao Y., Jin H., Slattery A. D., Davey K., Wang H., Qiao S. Z. (2018). 2D MoN-VN Heterostructure ToRegulate
Polysulfides for Highly Efficient Lithium-Sulfur Batteries. Angew. Chem., Int. Ed..

[24] Fan Q., Liu W., Weng Z., Sun Y., Wang H. (2015). Ternary Hybrid Material
for High-Performance Lithium-Sulfur Battery. J. Am. Chem. Soc..

[25] Zhang W. X., Kong S. L., Wang W. W., Cheng Y. M., Li Z., He C. (2025). Enhanced Electrocatalytic
Performance of LCO-NiFe-C3N4 Composite
Material for Highly Efficient Overall Water Splitting. J. Colloid Interface Sci..

[26] Zhai S., Liu W., Hu Y., Chen Z., Xu H., Xu S., Wu L., Ye Z., Wang X., Mei T. (2022). Kinetic Acceleration
of Lithium Polysulfide Conversion via a Copper-Iridium Alloying Catalytic
Strategy in Li-S Batteries. ACS Appl. Mater.
Interfaces.

[27] Zhou T., Lv W., Li J., Zhou G., Zhao Y., Fan S., Liu B., Li B., Kang F., Yang Q. H. (2017). Twinborn TiO2-TiN
Heterostructures Enabling Smooth Trapping-Diffusion-Conversion of
Polysulfides towards Ultralong Life Lithium-Sulfur Batteries. Energy Environ. Sci..

[28] Li X., Guo G., Qin N., Deng Z., Lu Z., Shen D., Zhao X., Li Y., Su B. L., Wang H. E. (2018). SnS2/TiO2
Nanohybrids Chemically Bonded on Nitrogen-Doped Graphene for Lithium-Sulfur
Batteries: Synergy of Vacancy Defects and Heterostructures. Nanoscale.

[29] Zhang Y., Zhang R., Guo Y., Li Y., Li K. (2024). A Review on
MoS2 Structure, Preparation, Energy Storage Applications and Challenges. J. Alloys Compd.

[30] Wang X., Zhang G., Wang B., Wu Y., Guo S. (2024). Micro-Nanostructure
Designed CoP@MoS2 Accelerating Polysulfide Conversion and Reaction
Kinetics for Lithium-Sulfur Battery. ACS Sustainable
Chem. Eng..

[31] Öztürk O., Gür E. (2024). Layered Transition Metal Sulfides for Supercapacitor
Applications. ChemElectroChem.

[32] Zeng P., Zhou Z., Li B., Yu H., Zhou X., Chen G., Chang B., Chen M., Shu H., Su J., Wang X. (2022). Insight into the Catalytic Role of
Defect-Enriched
Vanadium Sulfide for Regulating the Adsorption-Catalytic Conversion
Behavior of Polysulfides in Li-S Batteries. ACS Appl. Mater. Interfaces.

[33] Fairley N., Fernandez V., Richard-Plouet M., Guillot-Deudon C., Walton J., Smith E., Flahaut D., Greiner M., Biesinger M., Tougaard S., Morgan D., Baltrusaitis J. (2021). Systematic
and Collaborative Approach to Problem Solving Using X-Ray Photoelectron
Spectroscopy. Appl. Surf. Sci. Adv..

[34] Kohn W., Sham L. J. (1965). Self-Consistent
Equations Including Exchange and Correlation
Effects. Phys. Rev..

[35] Blochl P. E. (1994). Projector
Augmented-Wave Method. Phys. Rev. B.

[36] Kresse G., Furthmü J. (1996). Efficient Iterative Schemes for Ab Initio Total-Energy
Calculations Using a Plane-Wave Basis Set. Phys.
Rev. B.

[37] Perdew J. P., Burke K., Ernzerhof M. (1996). Generalized
Gradient Approximation
Made Simple. Phys. Rev. Lett..

[38] Kresse G., Joubert D. (1999). From Ultrasoft Pseudopotentials to the Projector Augmented-Wave
Method. Phys. Rev. B.

[39] Monkhorst H. J., Pack J. D. (1976). Special Points for
Brillonin-Zone Integrations. Phys. Rev. B.

[40] Grimme S. (2006). Semiempirical
GGA-Type Density Functional Constructed with a Long-Range Dispersion
Correction. J. Comput. Chem..

[41] Fan X. L., An Y. R., Guo W. J. (2016). Ferromagnetism
in Transitional Metal-Doped
MoS2Monolayer. Nanoscale Res. Lett..

[42] Mao Y., Bai J., Si J., Ma H., Li W., Wang P., Zhang H., Sheng Z., Zhu X., Tong P., Zhu X., Zhao B., Sun Y. (2023). Magneto-Electrochemistry-Driven
Ultralong-Life
Zn-VS2 Aqueous Zinc-Ion Batteries. Mater. Horiz..

[43] Wang V., Xu N., Liu J. C., Tang G., Geng W. T. (2021). VASPKIT: A User-Friendly
Interface Facilitating High-Throughput Computing and Analysis Using
VASP Code. Comput. Phys. Commun..

[44] Samad A., Shin Y. H. (2017). MoS2@VS2 Nanocomposite
as a Superior Hybrid Anode Material. ACS Appl.
Mater. Interfaces.

[45] Du J., Xia C., Xiong W., Wang T., Jia Y., Li J. (2017). Two-Dimensional
Transition-Metal Dichalcogenides-Based Ferromagnetic van Der Waals
Heterostructures. Nanoscale.

[46] Sun J., Dou H., Leng J., Zheng F., Zhang G. (2021). Modulation of the Contact
Barrier at VS2/MoS2 Interface: A First Principles Study. Phys. Lett. A.

[47] Mak K. F., Lee C., Hone J., Shan J., Heinz T. F. (2010). Atomically Thin
MoS2: A New Direct-Gap Semiconductor. Phys.
Rev. Lett..

[48] Niu Z., Feng T., Li T., Yang K., Zhao J., Li G., Li D., Zhao S., Qiao W., Chu H. (2021). Layered
Metallic Vanadium Disulfide for Doubly Q-Switched Tm: YAP
Laser with EOM: Experimental and Theoretical Investigations. Nanomaterials.

[49] Zhang S., Wang J., Torad N. L., Xia W., Aslam M. A., Kaneti Y. V., Hou Z., Ding Z., Da B. (2020). Rational Design of Nanoporous MoS2/VS2 Heteroarchitecture
for Ultrahigh
Performance Ammonia Sensors. Small.

[50] Xu R., Huang J., Cao L., Feng L., Feng Y., Kou L., Liu Q., Yang D., Feng L. (2020). VS2Microflowers with
In Situ Embedded Few-Layer MoS2 Nanobelts for Enhanced Hydrogen Evolution
Reaction at High Current Density. J. Electrochem.
Soc..

[51] Huang X., Wang Z., Knibbe R., Luo B., Ahad S. A., Sun D., Wang L. (2019). Cyclic Voltammetry in Lithium–Sulfur Batteries—Challenges
and Opportunities. Energy Technol..

[52] Liu G., Zeng Q., Sui X., Tian S., Sun X., Wu Q., Li X., Zhang Y., Tao K., Xie E. (2023). Modulating D-Band Electronic Structures of Molybdenum Disulfide via
p/n Doping to Boost Polysulfide Conversion in Lithium-Sulfur Batteries. Small.

