# Supplementary material for: Synergistic Effect of VS 2 /MoS 2 as an Electrocatalyst for Accelerating Polysulfide Conversion in Lithium–Sulfur Batteries
Source: ACS Appl Mater Interfaces. 2025 Sep 25;17(40):56153–63. doi: 10.1021/acsami.5c12948 (PMC12516672; doi:10.1021/acsami.5c12948)
Supplement: Supplementary file 1 [file am5c12948_si_001.pdf]

Supporting Information

Synergistic Effect of VS_2/MoS_2 as an Electrocatalyst for Accelerating Polysulfide Conversion of Lithium-Sulfur Batteries

Thilini Boteju[1], Abinaya Sivakumaran[1], Sathish Ponnurangam[2*], and Venkataraman Thangadurai[1,3*]

[1]Department of Chemistry, University of Calgary, Calgary, Alberta T2N 1N4, Canada

[2]Department of Chemical and Petroleum Engineering, University of Calgary, Calgary, Alberta T2N 1N4, Canada

[3]School of Chemistry, University of St Andrews, North Haugh St Andrews, KY16 9ST, United Kingdom

[*]E-mail: sathish.ponnurangam@ucalgary.ca; vt36@st-andrews.ac.uk

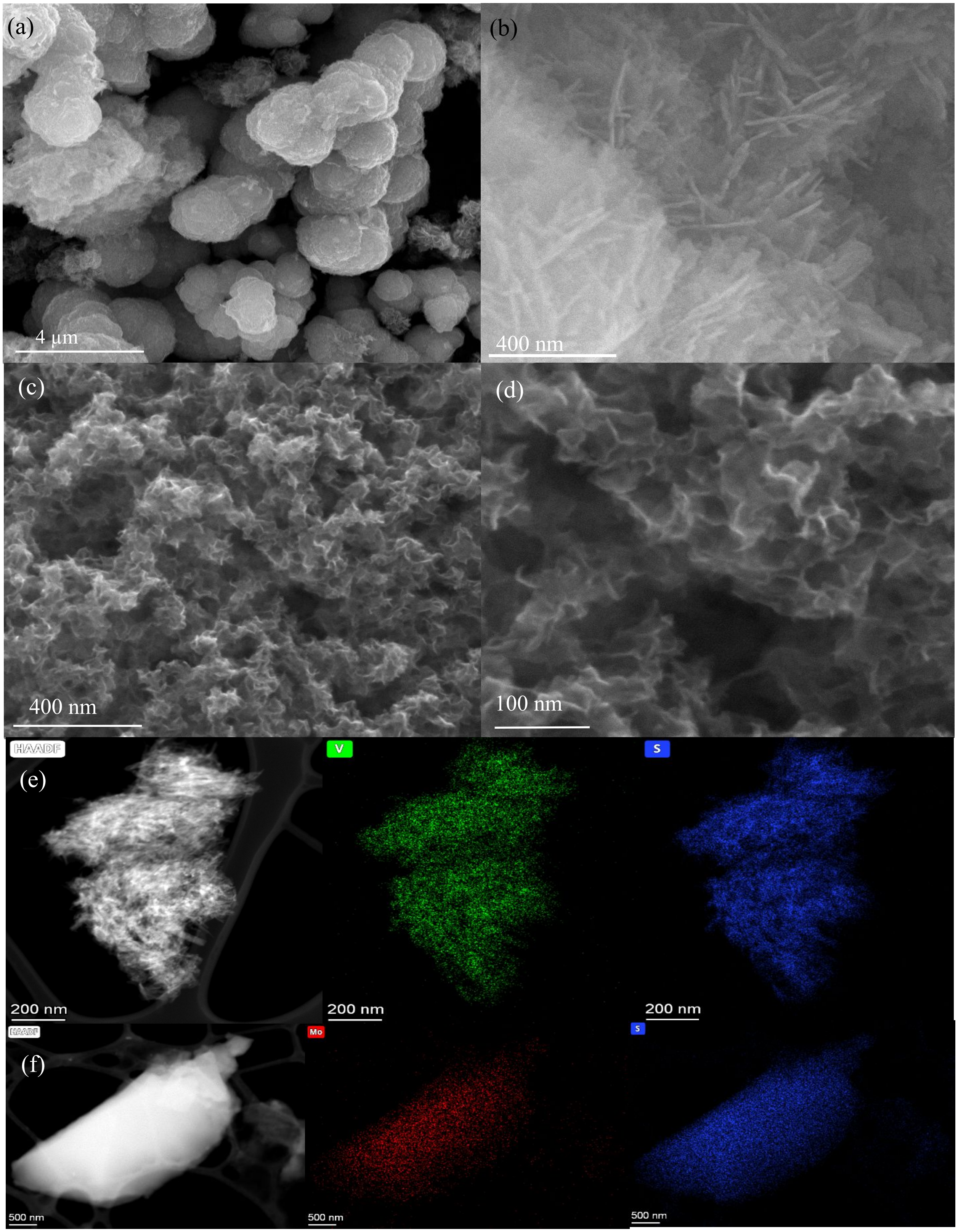

Figure S1: SEM images of pristine (a-b) VS_2 (c-d) MoS_2, EDX of pristine (e) VS_2 (f) MoS_2

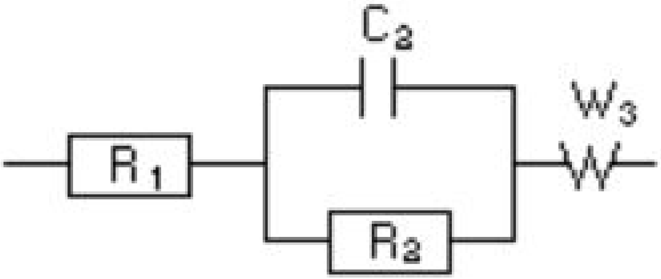

Figure S2: Schematic representation of the equivalent circuit diagram utilized in EIS.

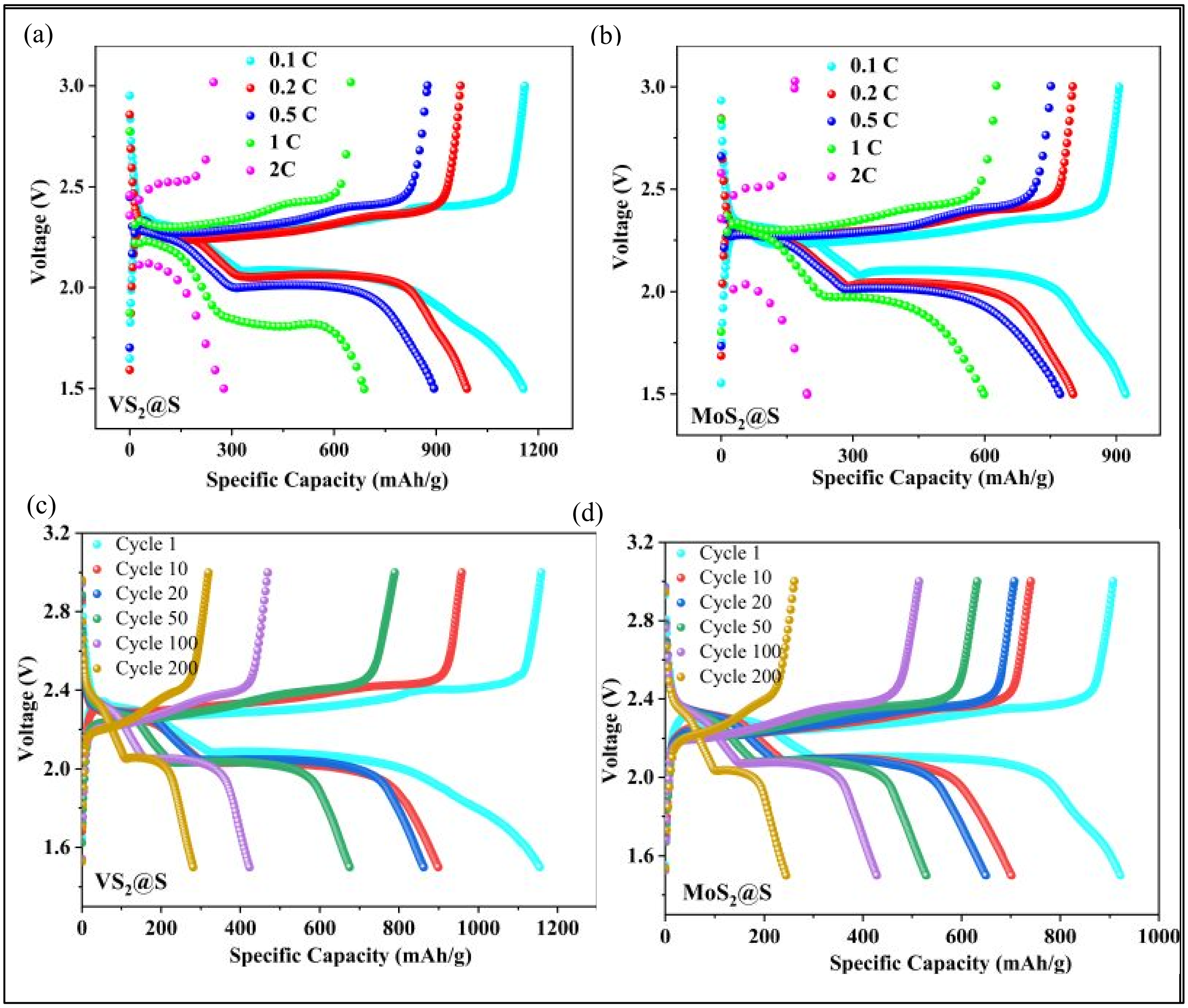

Figure S3: Discharge-charge profile for (a) VS_2@S (b) MoS_2@S at different C-rates and (c) VS_2@S (d) MoS_2@S cathode from cycle 1 to 200 at 0.1 C.

Table S1. The EIS fitting parameters of different cathode lithium-sulfur batteries.

Cathode	R_s (Ω)	R_{ct} (Ω)
VS_2@S	7.623	20.11
MoS_2@S	4.837	29.68
VS_2/MoS_2@S	3.079	15.8

Table S2. Comparison of the electrocatalytic performance of VS_2/MoS_2 with other transition metal-based catalysts reported recently.

Electrocatalyst	S loading (mg cm^{-2})	C-rate	Discharge capacity (mAh g^{-1})	Decay rate per cycle (mAh g^{-1})	Ref
VS_2/MoS_2	1.7	0.2	721/500	0.08 %	This work
CoP@MoS_2	2	0.2	745/300	0.12 %	[1]
Ni-MoS_2/rGO	1	1	422/400	0.11 %	[2]
rGO-MoS_2 QDs	1.3-1.5	2	503/300	0.11 %	[3]
Ni_3S_2-NiO	3	1	584/200	0.11 %	[4]
TiO_2@g-C_3N_4/CNT	2.58	1	569/800	0.07 %	[5]
MoS_2/PSN	1.2	2	521/600	0.056 %	[6]
MCNT	1.2	1	440/660	0.075 %	[7]

References

(1) Wang, X.; Zhang, G.; Wang, B.; Wu, Y.; Guo, S. Micro-Nanostructure Designed CoP@MoS2 Accelerating Polysulfide Conversion and Reaction Kinetics for Lithium-Sulfur Battery. *ACS Sustain Chem Eng* **2024**. https://doi.org/10.1021/acssuschemeng.4c04968.

(2) Zhang, R.; Dong, Y.; Al-Tahan, M. A.; Zhang, Y.; Wei, R.; Ma, Y.; Yang, C.; Zhang, J. Insights into the Sandwich-like Ultrathin Ni-Doped MoS2/RGO Hybrid as Effective Sulfur Hosts with Excellent Adsorption and Electrocatalysis Effects for Lithium-Sulfur Batteries. *Journal of Energy Chemistry* **2021**, *60*, 85–94. https://doi.org/10.1016/j.jechem.2021.01.004.

(3) Wei, H.; Ding, Y.; Li, H.; Zhang, Q.; Hu, N.; Wei, L.; Yang, Z. MoS2 Quantum Dots Decorated Reduced Graphene Oxide as a Sulfur Host for Advanced Lithium-Sulfur Batteries. *Electrochim Acta* **2019**, *327*. https://doi.org/10.1016/j.electacta.2019.134994.

(4) Du, X.; Ma, Y.; Zhang, W.; Zhang, M.; Su, K.; Li, Z. The Collaborative Effect of Ni3S2-NiO Heterojunction and Porous Carbon Network Modified Lithium-Sulfur Battery Separator for Effectively Inhibiting Polysulfides Shuttle. *J Power Sources* **2024**, *623*. https://doi.org/10.1016/j.jpowsour.2024.235414.

(5) Dong, L.; Jiang, W.; Pan, K.; Zhang, L. Rational Design of TiO2@g-C3N4/CNT Composite Separator for High Performance Lithium-Sulfur Batteries to Promote the Redox Kinetics of Polysulfide. *Nanomaterials* **2023**, *13* (24). https://doi.org/10.3390/nano13243084.

(6) Zheng, M.; Guo, C.; Luo, Z.; Wu, J.; Tang, X.; Li, L.; Sun, Q.; Ouyang, Q.; Shi, B.; Nie, H.; Shao, J. J.; Zhou, G. Molybdenum Disulfide (MoS2)/Porous Silica Nanosheet Composite Barrier for Polysulfide Shuttling Inhibition in Lithium-Sulfur Batteries. *Compos B Eng* **2023**, *264*. https://doi.org/10.1016/j.compositesb.2023.110898.

(7) Zheng, M.; Luo, Z.; Song, Y.; Zhou, M.; Guo, C.; Shi, Y.; Li, L.; Sun, Q.; Shi, B.; Yi, Z.; Su, F.; Shao, J.; Zhou, G. Carbon-Coated Nitrogen, Vanadium Co-Doped MXene Interlayer for Enhanced Polysulfide Shuttling Inhibition in Lithium-Sulfur Batteries. *J Power Sources* **2023**, *580*. https://doi.org/10.1016/j.jpowsour.2023.233445.

****
